# *ADIPOQ* single nucleotide polymorphism: Association with adiponectin and lipoproteins levels restricted to men

**DOI:** 10.1016/j.mgene.2015.06.003

**Published:** 2015-06-17

**Authors:** Luciane Viater Tureck, Neiva Leite, Ricardo Lehtonen Rodrigues Souza, Luciana da Silva Timossi, Ana Claudia Vecchi Osiecki, Raul Osiecki, Lupe Furtado Alle

**Affiliations:** a Polymorphism and Linkage Laboratory, Department of Genetics, Federal University of Paraná, Brazil; b Department of Physical Education, Federal University of Paraná, Brazil

**Keywords:** AMPK, adenosine monophosphate-activated protein kinase, BMI, body mass index, ELISA, enzyme-Linked Immunosorbent assay, GWA study, genome-wide association study, HL, hepatic lipase, HDL-C, high density lipoprotein cholesterol, IDL, intermediate density lipoproteins, LPL, lipoprotein lipase, LDL-C, low density lipoprotein cholesterol, LDLR, LDL-C receptor, SNP, single-nucleotide polymorphism, TC, total cholesterol, TG, triglycerides, VLDL, very low density lipoproteins, 276G > T SNP, *ADIPOQ* gene, Adiponectin, Lipid metabolism, Gender effect

## Abstract

Adiponectin is an adipokine inversely correlated with obesity, which has beneficial effect on insulin resistance and lipid metabolism. Considering its potential as a therapeutic target in the metabolic disorder contexts, and in order to add knowledge in the area, our study evaluated the *ADIPOQ* 276G > T polymorphism effect on adiponectin levels, and on lipoproteins of clinical interest in a population sample composed of 211 healthy individuals. Significant effects were observed only among men: the carriers of heterozygous genotype (GT) showed high levels of adiponectin (p = 0.018), while the rare homozygous genotype (TT) gave its carriers a negative phenotype, represented by higher levels of low density lipoprotein cholesterol (LDL-C) (p = 0.004 and p = 0.005) and total cholesterol (TC) (p = 0.010 and p = 0.005) compared to carriers of other genotypes (GG and GT respectively), the independent effect of SNP on LDL-C and TC levels was confirmed by multiple regression analysis (p = 0.008 and p = 0.044). We found no evidence of correlation between circulating adiponectin levels and biochemical markers, which suggests, therefore, an SNP 276G > T independent effect on adiponectin levels and on lipoprotein metabolism in men enrolled in this study.

## Highlights

• Gender-dependent association of *ADIPOQ* 276G > T SNP.
• Heterozygous men had higher levels of circulating adiponectin.
• Rare homozygous men had higher TC and LDL-C levels.

## Introduction

Adipose tissue, besides the traditional functions of energy storage and thermal insulator, is considered as an endocrine organ (Kershaw and Flier, 2004), able of acting in multiple metabolic pathways, due large amounts of bioactive compounds that are secreted by it, including free fatty acids and various adipokines, such as adiponectin (Lara-Castro et al., 2007, Whitehead et al., 2006).

Adiponectin is exclusively secreted by adipose tissue, being inversely correlated with obesity (Arita et al., 1999). Systemic action of adiponectin has been demonstrated, contributing for the glucose and lipid metabolism modulation (Cnop et al., 2003, Zhao and Zhao, 2011), with anti-inflammatory and antiatherogenic properties (Kubota et al., 2002, Nawrocki and Scherer, 2004). It acts through activation of several intracellular pathways, including the adenosine monophosphate-activated protein kinase (AMPK) pathway, promoting the free fatty acids oxidation and increasing glucose uptake by reducing gluconeogenesis in the liver (Fruebis et al., 2001). These actions are associated with improvement of insulin resistance markers (Hotta et al., 2000, Weyer et al., 2001), increasing of nitric oxide production (Chen et al., 2003, Qiao et al., 2008), and suppression of endothelial expression of adhesion molecules, smooth muscle cells proliferation, and the transformation of macrophages into foam cells (Ouchi et al., 1999).

The variants of adiponectin gene (*ADIPOQ*; 3q27.3) were associated with obesity, metabolic syndrome markers and cardiovascular disease (Cheung et al., 2014, Gao et al., 2013, Lu et al., 2014). Given the complex network of adiponectin interactions with several enzymes and hormones, and a wide range of modulator factors, the study's results which evaluate the *ADIPOQ* single-nucleotide polymorphisms (SNPs) effects on metabolism homeostasis are quite diverse (Gao et al., 2013, Zhao and Zhao, 2011), but very promising (Cheung et al., 2014, Lu et al., 2014), revealing that some variants seem to play a relevant role in these contexts.

Considering the adiponectin functional role in the metabolic processes of various clinical contexts, and its possibility to become a therapeutic target (Caselli, 2014, Hand et al., 2015), our work aims to contribute to knowledge in the area by evaluating the variant 276G > T (rs1501299) effect on adiponectin levels, and on biochemical markers associated with metabolic syndrome and cardiovascular risk in a population sample from Southern Brazil. The 276G > T SNP was chosen for this study because it had previously been associated with changes in the adiponectin levels (Heid et al., 2010, Ramya et al., 2013) and traits related to obesity (Lu et al., 2014) and metabolic syndrome (Ouyang et al., 2014), furthermore, the 276G > T effect on biochemical variables had not been investigated in individuals from Southern Brazil.

## Materials and methods

### Subjects

The sample consisted of 211 workers of Euro–Brazilian descent, employed by the Federal University of Paraná in Southern Brazil. Since the aim in selecting the volunteers was to obtain a sample representative of the population heterogeneity, no pathology was used as an inclusion or exclusion criterion. The study was approved by the ethics committee of the Federal University of Paraná (CEP/SD 1159.084.11.06/CAAE0082.0.091.000-11), and the study time period was the year 2012–2014.

Of these 211 individuals, 137 are woman, aged between 24 and 70 years old, and mean BMI = 27.20 (60.9% showed overweight and obesity). The Men (N = 74) aged between 26 and 59 years old, and mean BMI = 27.02 (66.6% exhibited overweight and obesity). Weight and height were measured with an accuracy of 0.1 kg and 0.1 cm respectively (BMI ≥ 25 = overweight; BMI ≥ 30 = obese).

The physical activity of the individuals was measured for seven days using a pedometer (YamaxDigi-Walker SW-700). This data showed that 23% were sedentary, 37% had low physical activity, 26% were active and 14% had high physical activity according to criteria proposed by Wyatt et al. (2005) and Tudor-Locke et al. (2011).

The sample used for independently test our results consisted of 182 premenopausal women from a different sample of a Southern Brazilian population.

### Biochemical parameters

Fasting glucose, triglycerides (TG), total cholesterol (TC) and high density lipoprotein cholesterol (HDL-C) were measured by standard automated methods. Low density lipoprotein cholesterol (LDL-C) levels were calculated using the Friedewald equation (Friedewald et al. 1972), for triglycerides levels below 200 mg/dL. In cases where this criterion was not observed, the LDL-C levels were quantified by direct assay.

Due to technical limitations, the adiponectin concentration was measured in a subgroup of 128 individuals randomly chosen from the total sample by Enzyme-Linked Immunosorbent Assay (ELISA) method, applied according to manufacturer's protocol (kit Elisa Duo Set Human Adiponectin, R&D systems).

### DNA analysis

DNA was extracted from peripheral blood by a salting-out method (Lahiri and Nurnberger Jr, 1991) and then diluted to a final concentration of 20 ng/μL. The intronic variant rs1501299 (276G > T) of *ADIPOQ* gene, were genotyped using a TaqMan allelic discrimination assay on an StepOnePlus™ real time PCR systems (Applied Biosystems, Foster City, CA, USA). The conditions of TaqMan reactions were as follows: 50 °C for 2 min, 95 °C for 10 min and 50 cycles of 95 °C for 15 s and 62 °C for 1 min. Three previously sequenced control samples, representative of each of the possible genotypes, were included in each reaction.

### Statistical analysis

Allele and genotype frequencies of the SNP genotyped were obtained by direct counting and Hardy–Weinberg equilibrium was tested with *χ*^2^ test.

The comparisons between means were performed by Mann–Whitney test, and within-group and between-group differences were analyzed using Kruskall–Wallis.

A multiple regression analysis was performed to confirm the hypothesis that the variables are independent factors for observed differences. Spearman rank order correlations analysis, for non-parametric correlation, was applied to test correlations between variables.

The probability value for the comparative tests were considered significant at p < 0.05 (5%).

## Results

The genotype distribution of the investigated SNP is in Hardy–Weinberg equilibrium, and allele and genotype frequencies are shown in Table 1.

The Kruskal–Wallis test results revealed differences in the levels of some biochemical variables: HDL-C, LDL-C and TC (p = 0.0379; p = 0.0133 and p = 0.0097 respectively) when compared carriers of the three possible SNP 276G > T genotypes (GG, GT, TT) of the *ADIPOQ* gene, and thus grouped only by genotype.

In order to identify where these differences lie, the three possible SNP 276G > T genotypes were compared two by two (Table 2), also having as grouping criterion only genotype. The tests results showed that homozygotes for the rare allele had significantly higher means of LDL-C and TC compared with heterozygotes (p = 0.003 and p = 0.004, respectively), and also when compared with common homozygotes (p = 0.013 and p = 0.018, respectively). No significant differences were observed in adiponectin levels among carriers of the three possible genotypes in the total sample (p > 0.4) (Table 2).

Considering the variations in metabolic processes which are inherent to gender, we conducted the same analyses grouped by gender and genotype. We observed that the differences in LDL-C and TC mean levels remained only in men, and between the same genotypes: homozygotes for the rare allele had higher mean levels compared with heterozygotes (p = 0.005 and p = 0.005 respectively) and common homozygotes (p = 0.004 and p = 0.010 respectively). The adiponectin mean levels are higher in heterozygotes compared with common homozygotes (p = 0.018) only in men (Table 3). When we analyze the genotypes together, differences in adiponectin, TC and LDL levels remained, as shown in Table 4.

**Fig. 1:**
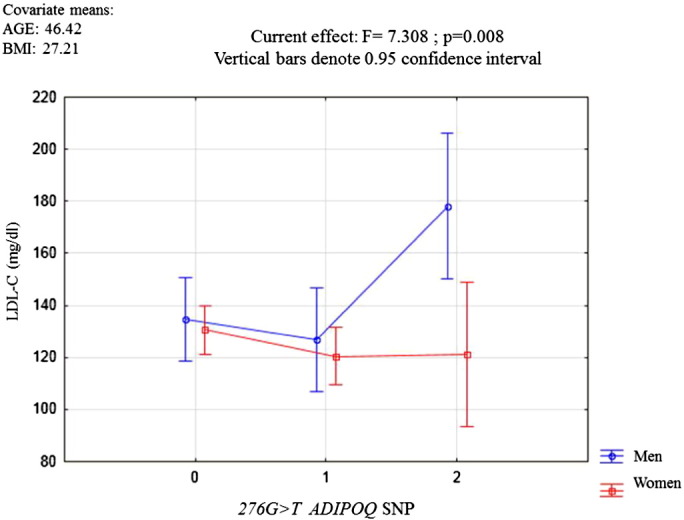
Multiple regression analysis, where the effect of the three possible 276G > T genotypes on LDL-C levels were evaluated in men and women. GG genotype (0); GT genotype (1), and TT genotype (2).

**Fig. 2:**
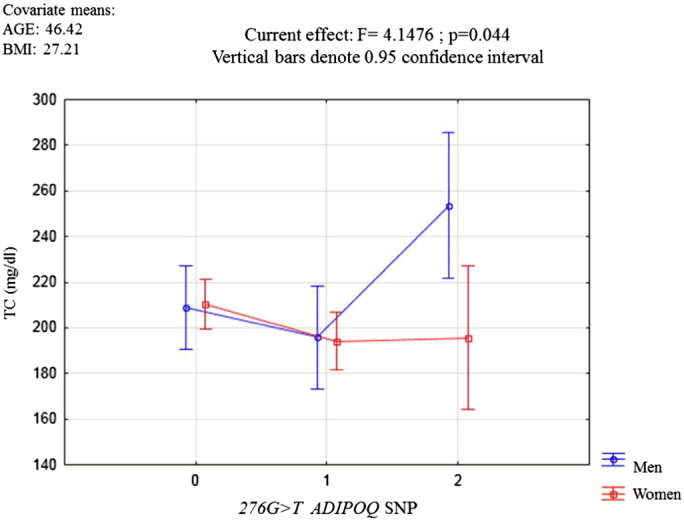
Multiple regression analysis, where the effect of the three possible 276G > T genotypes on TC levels were evaluated in men and women. GG genotype (0); GT genotype (1), and TT genotype (2).

Multiple regression analysis tests were performed to confirm the observed effect of the SNP 276G > T genotypes (independent variable), age and BMI (continuous variables/covariates) on the LDL-C and TC levels (dependent variables), categorizing the samples by gender. The results showed that the SNP 276G > T genotype acted independently for the determination of LDL-C and TC levels in men (F = 7.308, p = 0.008; and F = 4.1476, p = 0.044 respectively) Fig. 1, Fig. 2.

The Spearman correlation analysis was performed to check the inter-relationship among adiponectin levels, 276G > T variant, LDL-C and TC levels in men. There was no significant correlation between the LDL-C and TC levels and adiponectin circulating levels (ρ = − 0.024, p > 0.05; ρ = − 0.030, p > 0.05, respectively). However the 276G > T variant was significantly correlated with adiponectin levels in men (ρ = 0.428, p < 0.05).

To validate our findings, the same tests were applied to an independent sample available in our database composed of 182 healthy women. The results also showed no association of *ADIPOQ* SNP 276G > T with any of the biochemical variables evaluated (Supplementary material).

## Discussion

The purpose of the present study was to investigate the 276G > T *ADIPOQ* variant effects on biochemical indicators and plasma adiponectin levels in a sample of a Southern Brazilian population of self-declared European ancestrality. Our work consisted of a cross-section analysis which allowed the identification of important associations, where the modulation of observed effects was markedly dependent on the gender.

Despite the fact that no differences were found in adiponectin mean levels between men and women (4.41 ± 2.19 and 4.44 ± 1.99 p = 0.628, respectively), it is known that plasma levels of this protein exhibit sexual dimorphism (Arita et al., 1999), and our sample was not sufficiently large to observe this effect. Women have significantly higher serum concentrations than men (Haluzík et al., 2004) regardless of the fat mass amount and distribution (Cnop et al., 2003). Contributing to these differences several factors are found, including nutritional (Alsaleh et al., 2013), endocrine (Henneman et al., 2010), and environmental, such as levels of physical activity (Saunders at al., 2012). Despite the heritability of plasma adiponectin levels may reach 88% (Cesari et al., 2007), it still shows gender variations: a study found that in men the genetic component contribution responsible for the variance in adiponectin levels was 34%, while in women no evidence of heritability was found (Miljkovic-Gacica et al., 2007).

Due to the multifactorial nature that controls adiponectin serum levels, the reproducibility of the studies associating genetic variants to this feature is low. Our results suggest that the 276G > T SNP contributed for the adiponectin levels variation only among men, conferring to heterozygotes (GT) higher mean levels when compared to common homozygotes (GG). However, this finding should be interpreted with caution, since these results were derived from small sample analysis, which may cause instability in the statistical significance found. Even considering the above, the data indicates a possible effect that worth being investigated in larger samples. The association of this SNP with adiponectin levels have been established through Genome-Wide Association Studies (GWAs) (Heid et al., 2006a, Heid et al., 2006b, Heid et al., 2010, Pollin et al., 2005), and the association with traits related to metabolic syndrome, diabetes and atherogenic indicators shows heterogeneous results (Al-Daghri et al., 2012, Arikoglu et al., 2014, Kawai et al., 2013, Melistas et al., 2009 Pyrzak et al., 2013, Zhao and Zhao, 2011), probably due to multiple factors that influence its modulation, in addition to methodological differences between studies.

Some work signaled to sex dependent effects, for example, the extensive study by Cheung and collaborators, which found 276G > T SNP association with coronary heart disease, and the T allele with hipoadiponectinaemia only in men (Cheung et al., 2014). Our findings also suggest that the SNP has differential effect on adiponectin levels and the lipoprotein metabolism in men, considering the detrimental effect that the rare allele in homozygous (TT) has conferred to carriers, contributing independently to increased levels of TC and LDL-C compared to common homozygous genotypes (GG) and heterozygous (GT). This result corroborates with others that suggests significant gender effect on the association between this variant and metabolic disorders (Khabour et al., 2013), and highlights the importance of future studies that consider stratification by gender in their analysis.

The steroid hormones act in adiponectin regulation and may influence the underlying mechanisms to variation of effect observed between the sexes. It was shown that castrated experimental animals had increased adiponectin levels, whereas when under the effect of supplemental testosterone the adiponectin levels decreased, demonstrating the testosterone effects in reducing plasmatic adiponectin concentration (Nishizawa et al., 2002). In the same study it was observed that mRNA adiponectin levels were not affected by testosterone in vitro or in vivo, suggesting that the hormone may act on the secretory pathway, and not necessarily on the regulation of its nuclear production, but the exact dynamics of the androgens action in this setting remains unknown (Nishizawa et al., 2002).

In addition, the lipoproteins of lipid metabolism, and the enzymes that modulate it, also differ in their distribution and activity in a gender-dependant way, as they are regulated by steroid hormones (Nikkila et al., 1984, Taskinen and Kuusi, 1986, The Lipid Research Clinics Program Epidemiology Committee, 1979). In women, the hepatic lipase (HL) activity is around 60% to 70% of the activity found in men (Kuusi et al., 1989), besides lipoprotein lipase (LPL) higher activity compared to men (Nikkila et al., 1984, Taskinen and Kuusi, 1986). Early in life, before the sex hormones action, HDL-C is similar between the sexes, but after puberty, decreases in boys and remains unchanged in girls (The Lipid Research Clinics Program Epidemiology Committee, 1979). It is suggested that activation of HL by testosterone is responsible for the decline in HDL-C levels (Sorva et al., 1988), and this difference remains in adult life. On the other hand, the LDL-C is lower in women, presumably due to the action of estrogens on the increase in LDL-C receptor (LDLR) activity (Kovanen et al., 1979), since after menopause this difference disappears between the sexes (The Lipid Research Clinics Program Epidemiology Committee, 1979). Variations in lipid metabolism inherent to sex may also contribute to the heterogeneity and lack of reproducibility of studies investigating the effects of factors that interfere in energy homeostasis, among these, the effects of genetic variants in genes encoding proteins that modulate these key enzymes, such as *ADIPOQ*.

Despite that the *ADIPOQ* gene variants have been associated with dyslipidemia (Hotta et al., 2000, Ryo et al., 2004), it is not clear whether there is a direct relationship between low adiponectin levels and this disease (Lara-Castro et al., 2007).

Often the 276G > T SNP association with metabolic disorders is not related to differences in plasma adiponectin (Gable et al., 2006, Heid et al., 2006a, Heid et al., 2006b), suggesting that in some cases the effect of the genetic variant may be independent of the circulating levels of the protein, as proposed by Qi (Qi et al., 2005). Likewise we propose that the 276G > T variant impacted independently on adiponectin levels and on lipid metabolism in men who composed our study, since there was no significant correlation between adiponectin levels and biochemical markers (p > 0.05). Furthermore, the independence of these effects was also verified by disparity in genetic composition responsible for the observed differences: rare allele homozygotes (TT) had higher LDL-C and TC average levels (recessive effect), while on the adiponectin levels only individuals with heterozygous genotype (GT) showed increased serum levels (overdominance).

Despite the diversity of observed results, some mechanisms have been proposed to explain the adiponectin influence on lipid homeostasis. This adipokine acts on plasma lipoprotein levels altering the levels and activity of key enzymes of lipid metabolism, among them the LPL and HL, previously mentioned (Lara-Castro et al., 2007). LPL hydrolyzes triglycerides in chylomicrons and in the very low density lipoproteins (VLDL), releasing free fatty acids and giving rise to intermediate density lipoproteins (IDL). Part of the IDL is removed by the liver, but most of it is converted into LDL-C by HL (Kersten, 2014). Transgenic mouse over expressing adiponectin showed increased expression and activity of LPL in skeletal muscle, and increased VLDL hydrolysis, thus leading to the decreased in TG levels (Qiao et al., 2008). Similar results were also described by (Combs et al., 2004) where female mice super expressing adiponectin showed increased expression of LPL in white adipose tissue, and the same results were found in similar studies in humans (De Vries et al., 2005, Von Eynatten et al., 2004). The increase in VLDL hydrolysis leads indirectly to decreased levels of total cholesterol. On the other hand, low levels of adiponectin are correlated with increased HL (Schneider et al., 2005) and decreased LPL activities (Saiki et al., 2007), which may lead to higher levels of LDL-C and reduction in HDL-C levels due to the combined effects of these two enzymes (Lara-Castro et al., 2007).

Given the complex nature which controls the lipoprotein and adiponectin circulating levels, the effect of SNP investigated in this study represents a small portion of many factors that contribute to the mechanisms underlying of these differences. We can state some limitations of our study, among them the relatively small sample size, because larger samples could reveal undetected effects of genotypic and allelic composition and confirm the effects found in this study. The replication of the tests in an independent sample of men would be interesting to validate our results, as well as the application of this study in postmenopausal women, which could provide similar results to those seen in men. We used only a premenopausal women sample for tests replication, whose results were similar to those found in the women group who composed the original sample.

In conclusion we propose a gender dependent effect of the 276G > T polymorphism on adiponectin levels, TC and LDL-C, with the heterozygous genotype (GT) conferring higher levels of protein, while the rare homozygous genotype (TT) was associated with higher TC and LDL-C. It is also important to emphasize that the polymorphism influence on the levels of biochemical markers was independent of adiponectin circulating levels.

## Funding

Grants and scholarships were received from Coordenação de Aperfeiçoamento de Pessoal de Nível Superior (CAPES) and Araucaria Foundation.

## Conflict of interest

The authors declare no conflicts of interest.

The following are the supplementary data related to this article.

Supplementary material Means (± SE) of glucose, HDL-C, LDL-C, TG, TC and paired comparisons (p) among carriers of the three possible 276G>T SNP genotypes in independent sample of women (N=182).

## Figures and Tables

**Table 1:** Allele and genotype frequencies of 276G > T SNP (% ± standard error) in the total sample (n = 211).

	Allele frequencies		Genotype frequencies	
G	69.9	± 0.01	GG	48.34
T	30.1	± 0.01	TG	43.13
			TT	8.53

**Table 2:** Means (± SE) of insulin, glucose, HDL-C, LDL-C, TG, TC and adiponectin, and paired comparisons (p) among carriers of the three possible 276G > T SNP genotypes.

Biochemical parameters	SNP *276G* > *T* genotypes				
GG^A^ (n = 102)	GT^B^(n = 91)	TT^C^ (n = 18)	Comparisons	p
Insulin (μUI/mL)	10.71 ± 10.32	9.15 ± 4.64	8.00 ± 3.87	AXB	0.595
BXC	0.626
AXC	0.380
Glucose (mg/dl)	84.58 ± 13.15	85.30 ± 20.88	82.16 ± 8.38	AXB	0.813
BXC	0.627
AXC	0.854
HDL-C (mg/dl)	50.56 ± 13.48	48.99 ± 12.77	52.22 ± 11.58	AXB	0.435
BXC	0.160
AXC	0.431
LDL-C (mg/dl)	131.26 ± 31.48	126.83 ± 28.66	151.99 ± 41.12	AXB	0.358
**BXC**	**0.003**
**AXC**	**0.013**
TG (mg/dl)	133.92 ± 65.15	126.34 ± 65.25	142.67 ± 88.30	AXB	0.288
BXC	0.468
AXC	0.895
TC (mg/dl)	208.59 ± 34.71	199.64 ± 37.90	228.88 ± 42.79	AXB	0.158
**BXC**	**0.004**
**AXC**	**0.018**
Adiponectin (ng/mL)	GG (n = 69)	GT (n = 48)	TT (n = 11)		p
4.35 ± 1.86	4.69 ± 2.36	3.97 ± 1.84	AXB	0.583
BXC	0.436
AXC	0.650

Note: A corresponds to the GG genotype; B to GT genotype and C to TT genotype.

Bold numbers represent p values significant.

**Table 3:** Biochemical variables means (± SE) in men and women, stratified by 276G > T SNP genotypes and compared two by two (p).

Biochemical parameters	Men					Women				
GG^A^ (n = 33)	GT^B^ (n = 35)	TT^C^ (n = 6)	Comparisons	p	GG (n = 69)	GT (n = 55)	TT (n = 12)	Comparisons	p
Insulin (μUI/Ml)	8.93 ± 5.40	9.60 ± 4.49	10.04 ± 4.18	AXB	0.477	11.50 ± 11.85	9.10 ± 4.77	6.31 ± 2.90	AXB	0.351
BXC	0.874	BXC	0.207
AXC	0.558	AXC	0.075
Glucose (mg/dl)	84.94 ± 11.34	86.71 ± 7.05	88.33 ± 5.28	AXB	0.095	84.40 ± 14.00	84.65 ± 26.27	79.08 ± 8.05	AXB	0.332
BXC	0.897	BXC	0.578
AXC	0.192	AXC	0.279
HDL-C (mg/dl)	44.91 ± 10.33	42.88 ± 6.62	46.83 ± 11.18	AXB	0.492	53.26 ± 14.03	53.12 ± 14.11	54.92 ± 11.26	AXB	0.910
BXC	0.568	BXC	0.578
AXC	0.683	AXC	0.623
LDL-C (mg/dl)	131.46 ± 28.29	131.22 ± 25.70	181.73 ± 45.12	AXB	0.883	131.16 ± 33.09	124.71 ± 30.17	137.12 ± 31.06	AXB	0.314
**BXC**	**0.005**	BXC	0.086
**AXC**	**0.004**	AXC	0.253
TG (mg/dl)	147.64 ± 72.18	150.09 ± 77.65	188.83 ± 118.31	AXB	0.801	127.36 ± 60.97	112.45 ± 57.63	119.58 ± 62.78	AXB	0.143
BXC	0.671	BXC	0.713
AXC	0.360	AXC	0.632
TC (mg/dl)	205.97 ± 30.87	204.17 ± 30.64	256.00 ± 45.46	AXB	0.703	209.84 ± 36.55	197.89 ± 41.41	215.33 ± 35.90	AXB	0.154
**BXC**	**0.005**	BXC	0.116
**AXC**	**0.010**	AXC	0.253

	Men					Women				
	GG (n = 21)	GT (n = 11)	TT (n = 5)	Comparisons	p	GG (n = 48)	GT (n = 37)	TT (n = 6)	Comparisons	p

Adiponectin (ng/mL)	3.96 ± 2.18	5.64 ± 2.28	3.62 ± 0.77	**AXB**	**0.018**	4.52 ± 1.70	4.40 ± 2.34	4.258798	AXB	0.364
BXC	0.079	BXC	0.930
AXC	0.696	AXC	0.804

Note: A corresponds to the GG genotype; B to GT genotype and C to TT genotype.

Bold numbers represent p values significant.

**Table 4:** Biochemical variables means (± SE) in men and women, stratified by 276G > T SNP grouped genotypes and compared two by two (p).

Biochemical parameters	Men						Women					
GG (n = 33)	GT + TT (n = 41)	p	GG + GT (n = 68)	TT (n = 6)	p	GG (n = 69)	GT + TT (n = 67)	p	GG + GT (n = 124)	TT (n = 12)	p
Insulin (μUI/Ml)	8.93 ± 5.4	9.73 ± 7.2	0.403	9.17 ± 5.0	10.04 ± 4.1	0.635	11.50 ± 11.8	8.71 ± 4.6	0.176	10.45 ± 9.4	6.31 ± 2.9	0.106
Glucose (mg/dl)	84.93 ± 11.3	86.95 ± 6.7	0.065	85.85 ± 9.3	88.33 ± 5.2	0.458	84.40 ± 14.0	83.65 ± 24.0	0.231	84.52 ± 20.2	79.08 ± 8.0	0.373
HDL-C (mg/dl)	44.9 ± 10.3	43.46 ± 7.4	0.625	43.87 ± 8.6	46.83 ± 11.1	0.599	53.26 ± 14.0	53.45 ± 13.5	0.794	53.20 ± 14.0	54.92 ± 11.2	0.583
LDL-C (mg/dl)	131.46 ± 28.2	138.6 ± 33.8	0.535	**131.33** ± **26.7**	**181.73** ± **45.1**	**0.003**	131.16 ± 33.0	126.93 ± 30.4	0.621	128.30 ± 31.8	137.11 ± 31.0	0.141
TG(mg/dl)	147.64 ± 72.1	155.76 ± 84.0	0.628	148.90 ± 74.4	188.83 ± 118.3	0.482	127.36 ± 60.9	113.73 ± 58.1	0.154	120.75 ± 59.7	119.58 ± 62.7	0.921
TC(mg/dl)	205.97 ± 30.8	211.76 ± 37.4	0.728	**205.04** ± **30.5**	**256.00** ± **45.4**	**0.005**	209.84 ± 36.5	201.01 ± 40.7	0.392	204.54 ± 39.0	215.33 ± 35.9	0.61

	Men						Women					
	GG (n = 21)	GT + TT (n = 16)	p	GG + GT (n = 32)	TT (n = 5)	p	GG (n = 48)	GT + TT (n = 43)	p	GG + GT (n = 85)	TT (n = 6)	p

Adiponectin (ng/mL)	**3.96** ± **2.18**	**5.01** ± **2.13**	**0.043**	4.53 ± 2.32	3.62 ± 0.76	0.689	4.52 ± 1.70	4.38 ± 2.33	0.373	4.47 ± 1.98	4.26 ± 2.47	0.848

Bold numbers represent p values significant.
