# Supplementary material for: ADIPOQ single nucleotide polymorphism: Association with adiponectin and lipoproteins levels restricted to men
Source: Meta Gene. 2015 Jun 17;5:98–104. doi: 10.1016/j.mgene.2015.06.003 (PMC4484719; doi:10.1016/j.mgene.2015.06.003)
Supplement: Supplementary material — Means (± SE) of glucose, HDL-C, LDL-C, TG, TC and paired comparisons (p) among carriers of the three possible 276G>T SNP genotypes in independent sample of women (N=182). [file mmc1.docx]

**Supplementary Material**

Means (± SE) of glucose, HDL-C, LDL-C, TG, TC and paired comparisons (p) among carriers of the three possible 276G>T SNP genotypes in independent sample of women (N=182).

| Biochemical parameters | SNP *276G>T* Genotypes | | | |  |
| --- | --- | --- | --- | --- | --- |
|  | GG^A^ (n=84) | GT^B^ (n=89) | TT^C^ (n=9) | Comparisons | p |
| Glucose (mg/dl) | 88.41±14.64 | 90.31±21.51 | 89.77±6.99 | AXB | 0.483 |
|  |  |  |  | BXC | 0.385 |
|  |  |  |  | AXC | 0.209 |
| HDL-C (mg/dl) | 51.20±11.98 | 53.07±13.39 | 48.33±9.61 | AXB | 0.420 |
|  |  |  |  | BXC | 0.223 |
|  |  |  |  | AXC | 0.454 |
| LDL-C (mg/dl) | 110.71±31.14 | 110.35±27.80 | 108.86±27.26 | AXB | 0.912 |
|  |  |  |  | BXC | 0.735 |
|  |  |  |  | AXC | 0.870 |
| TG (mg/dl) | 146.09±83.86 | 130.40±52.65 | 147.33±50.21 | AXB | 0.410 |
|  |  |  |  | BXC | 0.304 |
|  |  |  |  | AXC | 0.447 |
| TC (mg/dl) | 191.19±37.12 | 189.51±35.44 | 186.66±37.16 | AXB | 0.762 |
|  |  |  |  | BXC | 0.819 |
|  |  |  |  | AXC | 0.729 |

Comparisons (p) between the parametric variables (TC) were performed by t-test; and between non-parametric variables (Glucose, HDL-C, LDL-C and TG) were performed by Mann-Whitney test
